# Identification of an immune-related six-long noncoding RNA signature as a novel prognosis biomarker for adenocarcinoma of lung

**DOI:** 10.1042/BSR20202444

**Published:** 2021-01-07

**Authors:** Huikai Miao, Dongni Chen, Rongzhen Li, Jia Hu, Youfang Chen, Chunmei Xu, Zhesheng Wen

**Affiliations:** 1 Department of Thoracic Oncology, State Key Laboratory of Oncology in South China, Collaborative Innovation Center for Cancer Medicine, Sun Yat-sen University Cancer Center, Guangzhou 510060, People’s Republic of China; 2 Department of Endocrinology and Metabology, Shandong Provincial Qianfoshan Hospital, Cheeloo College of Medicine, Shandong University, Jinan 250014, People’s Republic of China

**Keywords:** adenocarcinoma of lung, immune, long noncoding RNA, prognosis signature

## Abstract

**Background:** Lung adenocarcinoma (LUAD) is a heterogeneous disease with high mortality. Close attention has been paid to immunotherapy in LUAD treatment. However, immunotherapy has produced different therapeutic effects because of immune heterogeneity. Long noncoding RNAs (lncRNAs) are survival prognostic indicators with functions in the immune process. The present study was designed to examine the predictive power of immune-related lncRNAs in LUAD prognosis and investigated potential molecular mechanisms.

**Methods:** Transcriptome profiling and LUAD sample clinical information were retrieved from online database. The immune-related lncRNAs signature was identified by Cox regression. Survival analysis was used to verify the validity of the prognosis model. Then, possible biological functions were predicted and the abundance of infiltrating immune cells in LUAD samples were further analyzed.

**Results:** An immune-associated lncRNAs signature was established by combining six lncRNAs. Patients with LUAD were stratified into high- and low-risk groups using the six lncRNAs signature. Patients in different risk levels had significantly different prognoses (*P*<0.001), and the immune-associated lncRNAs signature was identified as an independent prognostic factor for LUAD. The functions of the lncRNA signature were confirmed as ubiquitin mediated proteolysis and signal sequence binding. The lncRNA signature negatively correlates with B-cell immune infiltration.

**Conclusion:** A reliable immune-related lncRNAs prognosis model for LUAD was identified. lncRNAs played a vital role in the tumor immune process and were associated with the LUAD prognosis. Research of lncRNAs in B-cell immune infiltration could provide new insight into the immunotherapy of LUAD.

## Introduction

Non-small cell lung cancer (NSCLC) is one of the most common carcinomas worldwide and imposes a major burden on health-care systems [1]. Adenocarcinoma of lung (LUAD) accounts for >40% of lung cancer cases, exceeding that of lung squamous cell carcinoma [1]. Due to its unclear histopathological behavior and metastatic character in an early stage, the prognosis for LUAD is generally poor. In recent years, a variety of genetic and immune microenvironmental factors have been shown to play significant roles in tumorigenesis and tumor progression [2]. In the process of LUAD development, tumor-induced immune suppression leads to imbalanced immune activity [3]. Based on the relevant immunological mechanisms that mediate tumor occurrence and development, several advances have been made in immunotherapy for LUAD [4], including immune checkpoint therapy and cancer vaccines. However, immune heterogeneity, including divergent immune profiles and immune cell repertoires in all patients [4], is associated with differential responses to immunotherapy [5].

Different genetic or molecular backgrounds generally lead to survival discrepancies. Regulators of gene expression, including long noncoding RNA (lncRNA), can mediate post-transcriptional modification and play key roles in immune response regulation. Therefore, tumor immunogenomics based research can expand our understanding of tumor immune mechanisms and precision medicine [6].

LncRNAs, noncoding RNAs with more than 200 nucleotides in length, are regarded as regulators in a wide range of biological functions [7]. LncRNAs regulate gene expression at the transcriptional, post-transcriptional, and epigenetic levels [8], which are involved in tumor suppression, tumorigenesis, and metastasis [9], and provide a new approach for genetic regulation of tumor development [10]. LncRNAs also have biological functions in tumor immunology, including tumor antigen presentation, immune escape, and immune cell infiltration [11]. LncRNAs are emerging as novel biomarkers for prognosis prediction in various cancers, including HOTAIR in lung cancer [12]. Many lncRNAs, including MALAT1 and MEG3, were reported to regulate the proliferation and metastasis of NSCLC [13,14], but the mechanism of immune balance mediated by lncRNAs in LUAD has been rarely studied. Based on the function of lncRNAs that participate in immune regulation, the obstacle of differential immunotherapeutic effects induced by immune heterogeneity needs to be solved. Therefore, the present study was designed to identify and verify a reliable lncRNA signature for the prognosis of LUAD. We also explored the role of lncRNAs in LUAD immune regulation using different bioinformatics methods.

## Materials and methods

### Workflow

**Figure 1:**
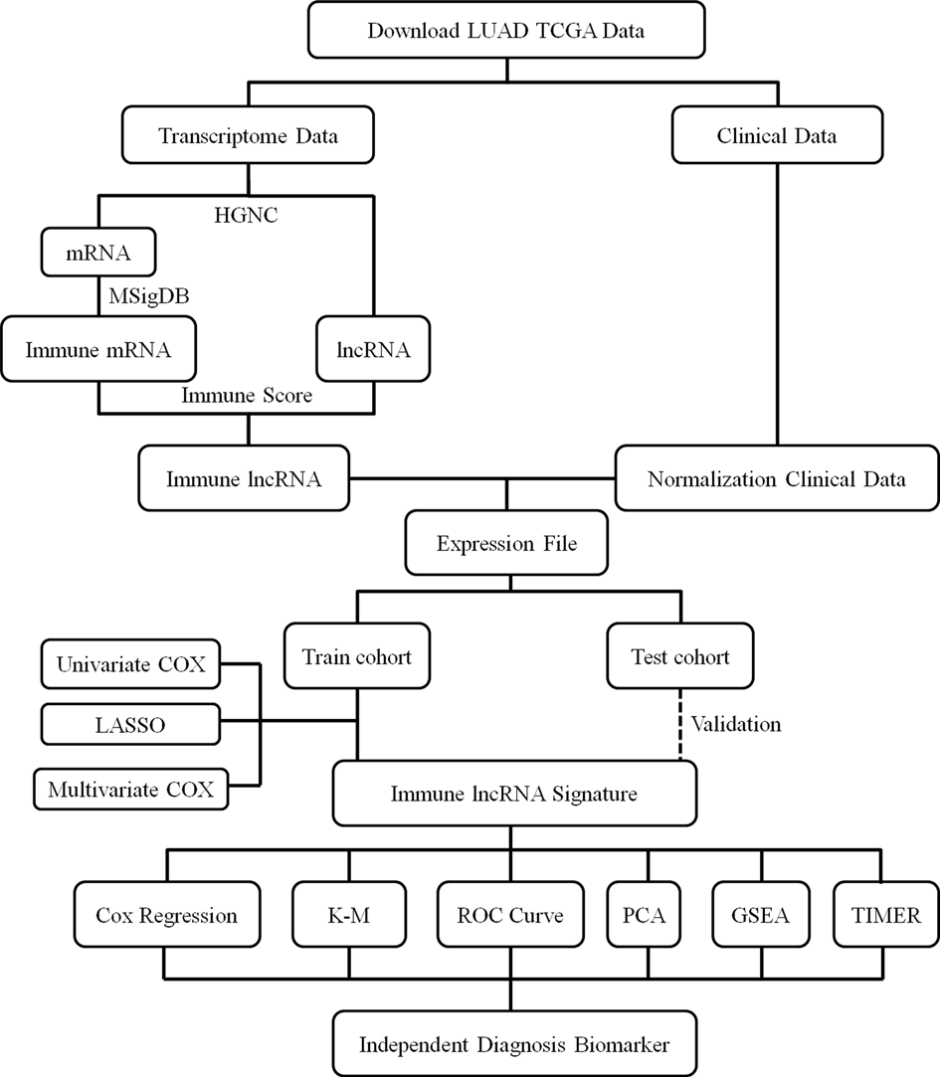
Research framework for the exploration procedure for the immune-related lncRNAs signature and the related mechanisms LUAD, Adenocarcinoma of Lung; TCGA, The Cancer Genome Atlas; HGNC, HUGO Gene Nomenclature Committee; mRNA, messenger RNA; lncRNA, long non-coding RNA; MSigDB, Molecular Signatures Database; LASSO, Least Absolute Shrinkage and Selection Operator; K-M, Kaplan–Meier; ROC, Receiver Operating Characteristic; PCA, Principal Component Analysis; GSEA, Gene Set Enrichment Analysis; TIMER, Tumor Immune Estimation Resource.

A multi-step approach was used to identify a multi-lncRNAs signature (Figure 1). The potential mechanisms through which these lncRNAs influence the prognosis of LUAD were further explored using The Cancer Genome Atlas (TCGA) project data. Univariate Cox analyses, multivariate Cox analyses, and least absolute shrinkage and selection operator (LASSO) method were applied to build a molecular prognostic model. This model graded patients into the high-risk and low-risk groups based on molecular score. Kaplan–Meier (K-M) analysis, receiver operating characteristic (ROC) curve, and principal component analysis (PCA) were conducted to analyze the survival prognosis of patients in different groups. High-risk LUAD patients showed significantly shorter survival time than did those designated low risk. The molecular signature was identified as an independent prognostic factor for LUAD by further multivariate Cox analysis in training and validation sets. Gene Set Enrichment Analysis (GSEA) was conducted to investigate the potential bio-function of the molecular signature. Immune cell infiltration abundance was calculated using the Tumor Immune Estimation Resource (TIMER) algorithm.

### Patients dataset processing

**Figure 2:**
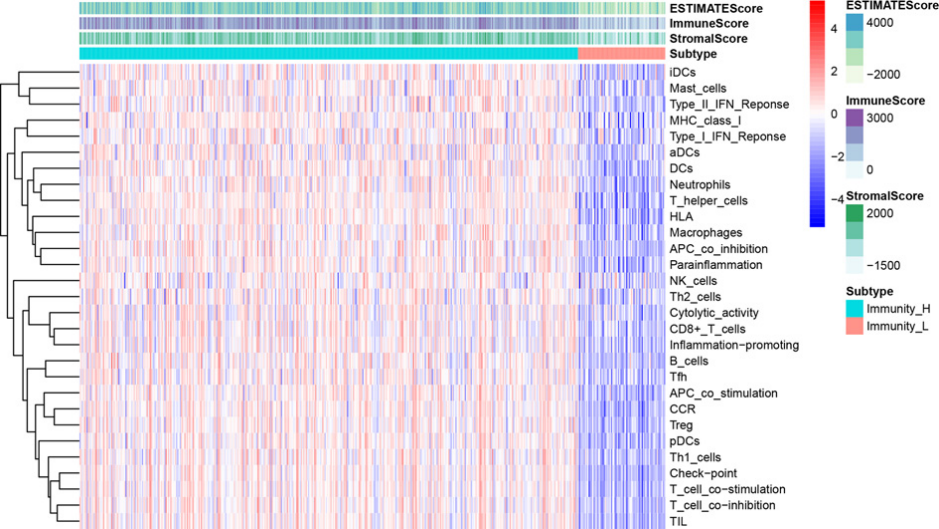
The enrichment levels of immune-associated gene sets and immune score of LUAD

LUAD transcriptome and clinical information were downloaded from the TCGA database. Patients with LUAD and insufficient prognostic data, such as incomplete survival information or follow-up time <30 days, were excluded. Overall survival (OS) was estimated as the primary endpoint. mRNAs and lncRNAs ensemble gene ids were derived from the HUGO Gene Nomenclature Committee (HGNC) database. Immune-related genes were obtained from the Molecular Signatures database (MSigDB) by downloading immune process and immune system response files. Twenty-nine immune-associated gene sets from ImmPort and Immunome Database were listed, representing diverse immune cell types, functions, and pathways. Single Sample Gene Set Enrichment Analysis (ssGSEA) was used to quantify the activity of enrichment levels of immune cells, functions, and pathways in each TCGA LUAD sample and to calculate the immune score. We divided the samples into high and low immunity groups using a clustering algorithm (Figure 2). Immune-related lncRNAs were identified by combining the expression of different lncRNAs and immune scores and determining their correlation. LUAD samples were divided into two cohorts by the ‘caret’ package of R software in a 5:5 ratio. These included a training cohort to identify lncRNAs associated with prognosis and built a prognostic risk model, and a testing cohort to validate the prognostic value of the risk model.

### Prognostic risk model and predictability evaluation

First, univariate Cox proportional hazards regression was applied to determine the association between immune-related lncRNA expression and OS in the training cohort. If the lncRNA *P* value was less than 0.01, they were selected as a candidate lncRNA significantly associated with prognosis. Then, we applied the LASSO method to filter optimal candidates. Multivariate Cox regression was performed among these LASSO candidates using the ‘survival’ and ‘survminer’ R packages. lncRNA candidates with corresponding *P* values less than 0.01 were considered as the key lncRNAs in the risk model. The risk score model for patients with LUAD was established as follows: Gene1 × Expression gene1 + Gene2 × Expression gene2 + Gene3 × Expression gene3 + … + Gene n × Expression gene n. Expression gene n was the expression value of each optimal prognostic lncRNA. Gene n was the regression coefficient of the multivariate Cox regression model for the target lncRNA.

We used the median risk score in the model as the cutoff value. Patients with LUAD were stratified into high- and low-risk groups by the risk score. K-M analysis was used to compare the survival rate with high-risk score indicative of poor survival. Then, we verified the reliability of the risk score formula on the testing cohort. Univariate and multivariate Cox regression analyses for OS were performed on the lncRNA signature together with other clinical risk factors. The hazard ratio (HR) and the 95% confidence interval (CI) were calculated. ROC curve analysis was conducted to evaluate the sensitivity and specificity of the risk model using the R package ‘survival ROC’. PCA of the lncRNAs model was completed for dimensionality reduction and quality control.

### Functional enrichment analysis

After identifying the lncRNA signature correlating with LUAD prognosis, the biological roles of the signature in gene ontology (GO) and Kyoto encyclopedia of genes and genomes (KEGG) pathways were assessed in DAVID database. These analyses were conducted for co-expressed protein-coding immune genes, using Pearson correlation coefficients (coefficients > 0.40, *P*<0.05) in our TCGA-LUAD cohort. *P* values were adjusted using Benjamini (NOM p-val) < 0.1, normalized enrichment score |NES| > 1.5 and false discovery rate (FDR q-val) < 0.05 were defined as threshold in GO and KEGG pathway categories using functional annotation chart options.

### Infiltration abundance analysis of immune cell

To further investigate the potential immunomodulatory mechanism of lncRNA in the regulation of tumor-infiltrating immune cells, the TIMER algorithm was used to assess the infiltration abundance of different immune cells in LUAD samples from TCGA. We analyzed the correlation between the abundance of immune cells and the lncRNA signature risk score. Then, we explored the relationship between the immune cell infiltration abundance and LUAD prognosis through univariate survival analysis. R software (version 3.6.3) and Perl 5 version 30 were used for all statistical analyses in the present study.

## Results

### Characteristics of the datasets

After detailed data processing, 445 patients with LUAD, complete lncRNA expression information, and clinical follow-up data were included. Patients were assigned into training (*n*=224) and testing (*n*=221) cohorts. The age of patients with LUAD ranged from 33 to 88 years old. Among them, 292 patients survived and 153 had died by the last follow-up. Survival post-diagnosis ranged from 34 to 6812 days.

### The identification of a lncRNA signature including six lncRNAs which were significantly associated with prognosis of LUAD

**Figure 3:**
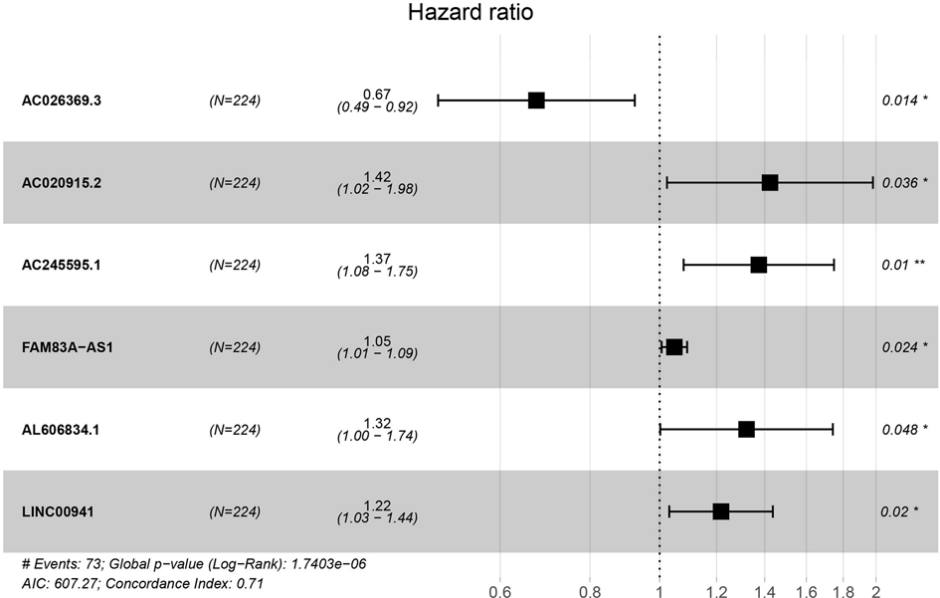
Forest map of the six-lncRNA signature

**Figure 4:**
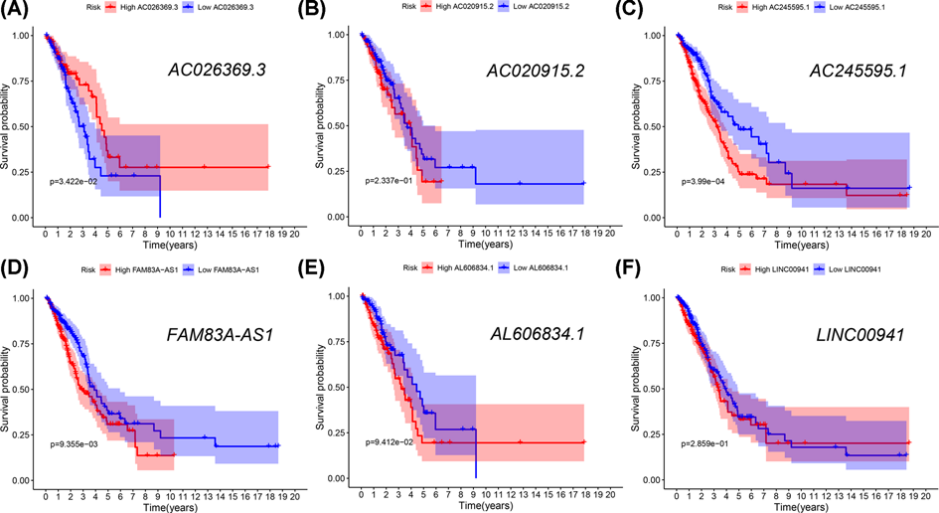
Kaplan–Meier analysis of the six lncRNAs The Kaplan–Meier analysis of the patients with high or low expression of AC026369.3 (**A**), AC020915.2 (**B**), AC245595.1 (**C**), FAM83A-AS1 (**D**), AL606834.1 (**E**), LINC00941 (**F**).

**Figure 5:**
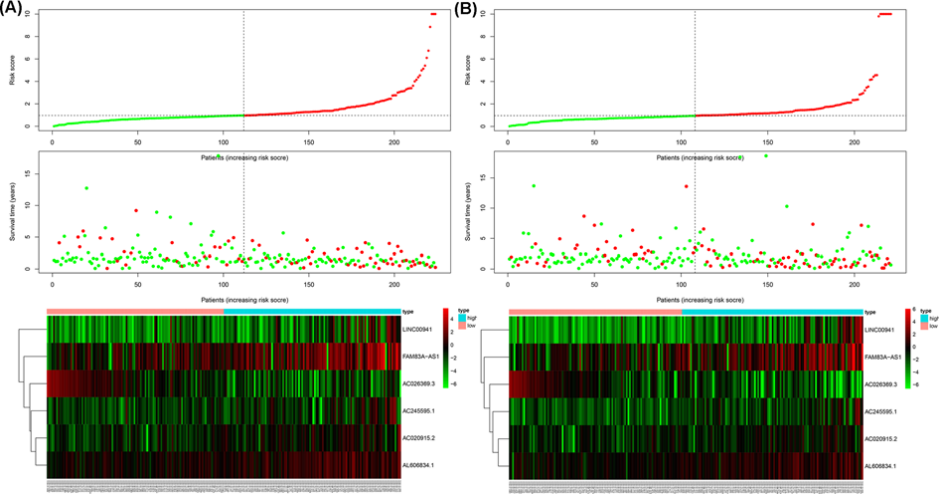
Risk score analysis of the six-lncRNA signature in LUAD patients Risk score analysis of the differentially expressed lncRNA signature of LUAD in the training cohort (**A**) and the testing cohort (**B**). Risk score of lncRNA signature (top); Survival status and duration of cases (middle); Low-score and high-score groups for the six lncRNAs (bottom).

**Figure 6:**
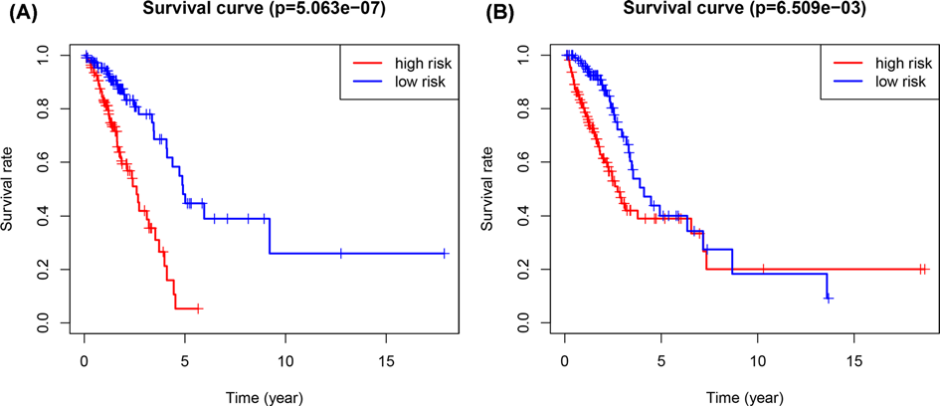
Kaplan–Meier test of the risk score The Kaplan–Meier test of the risk score for the overall survival in the training cohort (**A**) and the testing cohort (**B**).

**Figure 7:**
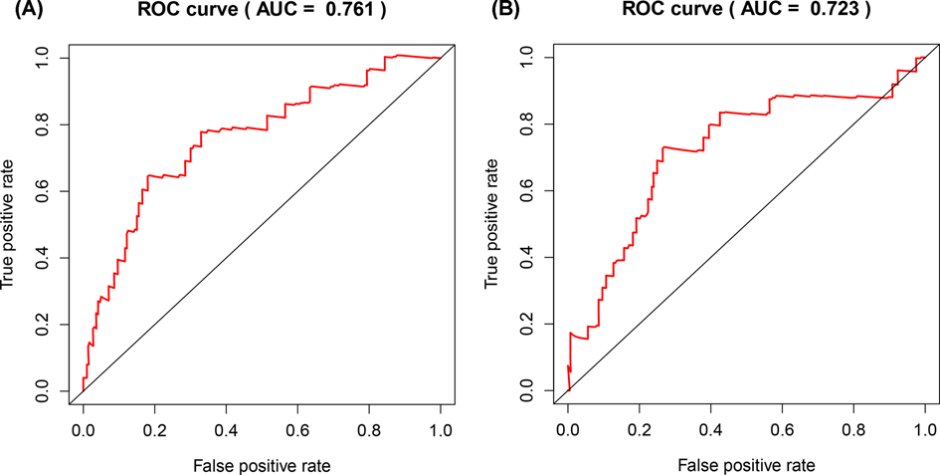
ROC curve of the risk score The risk score is shown by the receiver operating characteristic curve for predicting survival of the training cohort (**A**) and the testing cohort (**B**); ROC curve, receiver operating characteristic curve.

The profiles of immune-related lncRNAs were combined with TCGA survival data. Twelve candidate survival-related lncRNAs were preliminarily identified using univariate Cox regression analysis in the training cohort (adjusted *P*<0.01). LASSO coefficient profiles and a partial likelihood deviance plots are shown in Supplementary Figure S1, and seven lncRNAs were further selected from the 12 candidates. After multivariate Cox analysis, six lncRNAs were included in the risk-scoring model as independent prognostic factors (*P*<0.05, Figure 3). Among the six prognostic lncRNAs, five lncRNAs (AC020915.2, AC245595.1, FAM83A-AS1, AL606834.1, and LINC00941) worked as risk factors and their high expressions were correlated with shorter survival time. Only one lncRNA (AC026369.3) tended to be a prognostic protective factor with better survival (Figure 4). The prognosis of patients with LUAD was predicted by the expression level of the above lncRNAs in the six-lncRNA signature. The risk score was calculated using the regression coefficients of the multivariate Cox regression model. Risk score = 0.353 * Expression AC020915.2 + 0.317 * Expression AC245595.1 + 0.047 * Expression FAM83A-AS1 + 0.278 * Expression AL606834.1 + 0.196 * Expression LINC00941 – 0.394 * Expression AC026369.3. The risk score models are shown in Figure 5, and include the distribution of risk scores for each patient and the risk-related lncRNAs heatmap. The six-lncRNA prognostic risk score was calculated by the risk score model for each patient in the training and testing cohort. Patients with LUAD were divided into high-risk and low-risk groups based on the risk score median value. K-M analysis indicated that the OS of patients in the high-risk group was much lower than that in the low-risk group (training cohort: *P*<0.001, testing cohort: *P*<0.001, Figure 6). The sensitivity and specificity of the six lncRNA model was calculated by the area under ROC (training cohort: AUC = 0.761, testing cohort: AUC = 0.723), suggesting that our six-lncRNA signature was effective for predicting survival (Figure 7).

### The six-lncRNA signature was identified as an independence prognostic value

**Figure 8:**
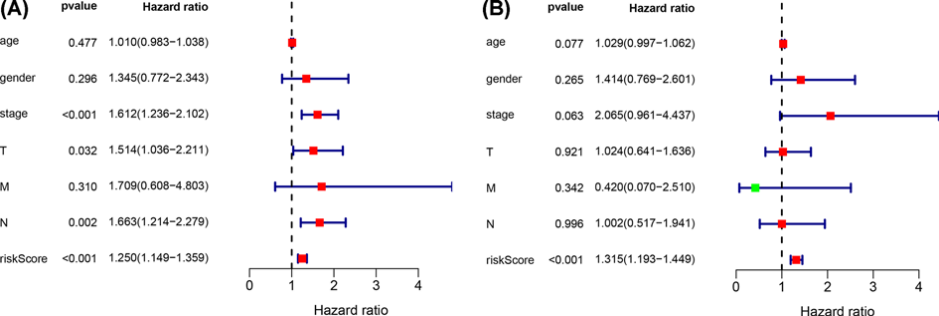
The predictive values of risk score in clinical features The predictive values of related clinical features and risk score by the univariate (**A**) and multivariate (**B**) Cox regression.

**Figure 9:**
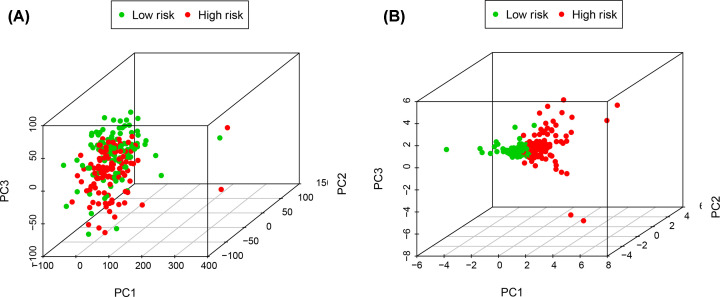
PCA analysis of the six-lncRNA signature PCA analysis of the independence of the immune-related lncRNA signature in all genes (**A**) and risk genes (**B**); PCA, principal component analysis.

We further evaluated whether the six-lncRNA signature showed an independent prognostic value compared to other risk factors. The univariate and multivariate Cox regression analyses were conducted based on the six-lncRNA signature and other clinicopathological variables, including age, gender, tumor size, TNM stage, lymph node metastasis, and distal metastasis (Figure 8). Tumor size (*P*=0.032), TNM stage (*P*<0.001), lymph node metastasis (*P*=0.002), and high-risk score (*P*<0.001) were significantly associated with poor OS. The risk score was defined as an independent prognostic factor for patients with LUAD (*P*<0.001). PCA revealed the independence of the immune-related lncRNA signature compared with other genes in LUAD patients (Figure 9).

### The six-lncRNA signature was involved in the ubiquitin signal pathway

**Figure 10:**
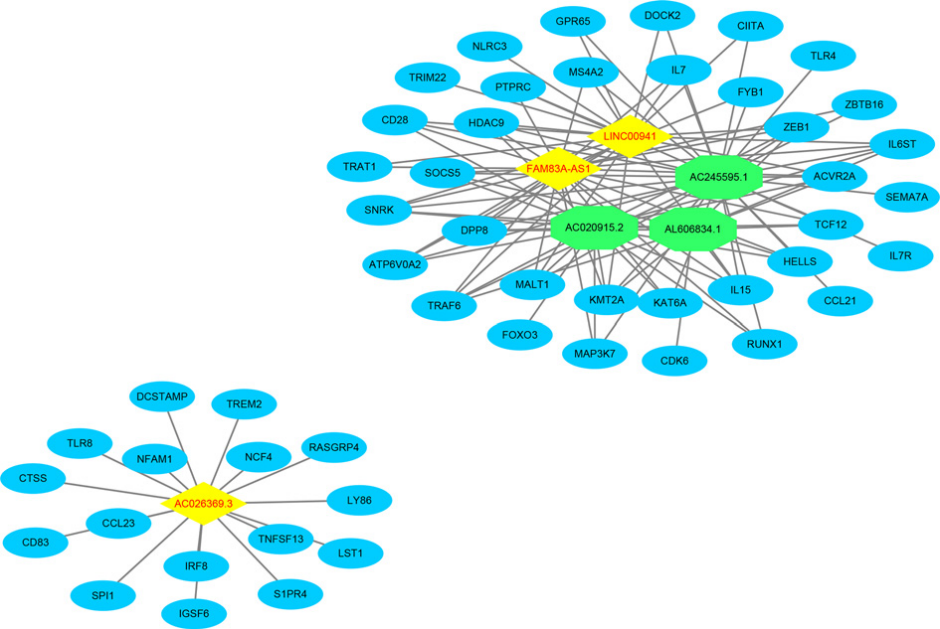
The network of the six lncRNAs signature and the related genes

**Figure 11:**
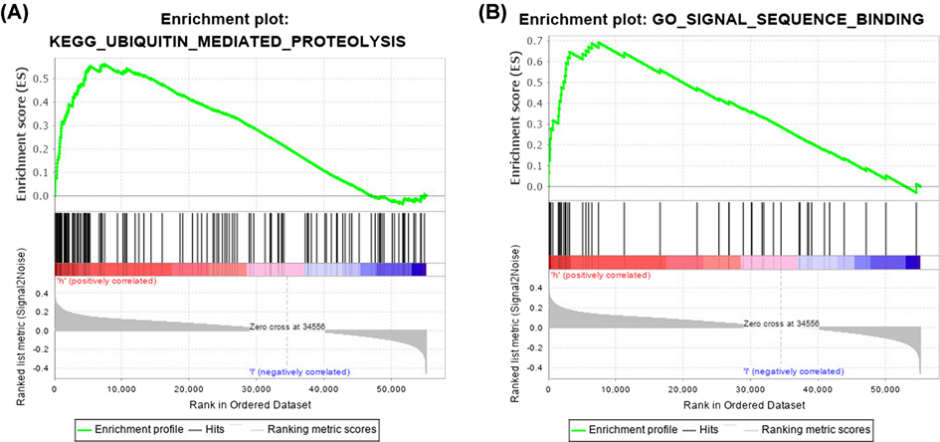
Score correlated enrichment gene analysis with GSEA (**A**) KEGG enrichment analysis showed that pathways associated with ubiquitin mediated proteolysis were significantly enriched in the high-risk group (NES = 2.07; NOM *P*-val = 0.000; FDR *q*-val = 0.017; FWER *P*-val = 0.019). (**B**) GO enrichment analysis showed that signal sequence binding was significantly enriched in the high-risk group (NES = 2.19; NOM *P*-val = 0.002; FDR *q*-val = 0.047; FWER *P*-val = 0.044); GSEA, Gene Set Enrichment Analysis; KEGG, Kyoto Encyclopedia of Genes and Genomes; GO, Gene Ontology; NES, normalized enrichment score; NOM *P*-val, adjusted *P* value by Benjamini; FDR *q*-val: adjusted *q* value by false discovery rate; FWER *P*-val: adjusted *P* value by Bonferonni.

We performed functional analysis of lncRNAs-related genes to clarify the potential biological roles of the six-lncRNA signature. The association of the six lncRNAs with these genes was visualized with Cytoscape software v3.6.1 (Figure 10). GO enrichment analysis showed that, in LUAD, the lncRNAs signature-related genes were mainly enriched in signal sequence binding. KEGG enrichment analysis revealed that these genes were mostly implicated in ubiquitin mediated proteolysis (Figure 11). These results indicated that the six lncRNAs might play a vital role in ubiquitin mediated proteolysis through mediating the downstream genes to participate in signal sequence binding processes.

### Expression of the six-lncRNA signature was related to tumor-infiltrating B cells

**Figure 12:**
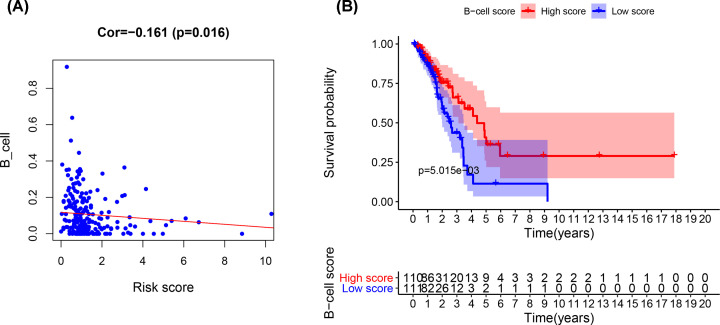
The survival analysis of B-cell infiltration (**A**) The co-expression between the lncRNA risk score and B-cell infiltration. (**B**) The survival analysis of B-cell infiltration.

To explore the relationship between the signature and tumor-infiltrating lymphocytes, we analyzed the abundance of six kinds of immune cells that infiltrated the tumor microenvironment. CD4 T cells, CD8 T cells, B cells, dendritic cells, macrophages, and neutrophils were assessed in LUAD samples from TCGA using the TIMER algorithm. We found that the abundance of infiltrating B cells was negatively related to our lncRNA risk score. Patients in high risk group had less B-cell infiltration than did patients in low risk group (Cor = –0.161, *P*=0.016, Figure 12A). Then, we investigated the association of immune cell infiltration with LUAD prognosis using K-M survival analysis. The results showed that the LUAD patients with high B-cell infiltration had better survival than those with low B-cell infiltration (*P*<0.001, Figure 12B).

## Discussion

LUAD is a common carcinoma and has the high morbidity and mortality in the world. Many patients are diagnosed with lung cancer in the advanced stages, which means that 5-year survival rate of patients with LUAD is very low [1]. Traditional treatments, such as surgery, radiotherapy, and chemotherapy, have limited effectiveness in some patients with LUAD because of recurrence and metastasis. Intra-tumor heterogeneity in individuals may lead to drug resistance and treatment failure [15]. The growth, invasion, and metastasis of LUAD often involves genetic abnormalities and immune dysfunction within the tumor microenvironment [16]. Given the important role of the immune system in tumor development [17], researchers have developed various immunotherapeutic strategies to treat tumors [4]. Monoclonal antibodies, such as programmed cell death 1 (PD-1) blockade and CTLA-4, have shown promising benefits in LUAD treatment by blocking immune checkpoint pathways [18]. However, the heterogeneity of morphologic and molecular characteristics of LUAD [19] means that individual patients have diverse responses to immunotherapy. Some patients with LUAD have an unfavorable prognosis and poor response to immunotherapy.

Molecular characterization has improved our understanding of lung cancer and highlights the need for novel molecular indicators for LUAD. The development of advanced sequencing technologies means that studies of genomic alterations in cancer cells are now common [20]. Large-scale consortia, such as TCGA, have contributed to the generation of vast amounts of genomic data [21]. Studies show that lncRNAs are involved in tumorigenesis and tumor suppression and are often regarded as powerful biomarkers for cancer diagnosis and prognosis and as targets for treatment [22,23]. Various lncRNAs modulate the immune system in LUAD [24]. More recently, lncRNA expression in tumors has been investigated in LUAD [25], but few studies have explored the immune regulation of lncRNA in LUAD.

To further explore the influence of lncRNA mediated immune regulation on LUAD prognosis, we focused on immune-related lncRNAs and built a prognostic formula with a good prognostic prediction for LUAD. Additionally, we chose multiple lncRNAs as prognosis signature for predictive accuracy and stability. Among the six identified lncRNAs, five (FAM83A-AS1, LINC00941, AC020915.2, AC245595.1, AL606834.1) were risk factors, and AC026369.3 was a protective factor. To further validate the model, we searched the six lncRNAs signature in many GSE datasets which were displayed in GEO database, a public functional genomics data repository. Whereas, due to the lack of detailed clinical data and insufficient newly identified lncRNAs in GEO database, we failed to find a perfect GEO dataset which included both the six lncRNAs expressions and reliable prognostic information. Previous studies found that FAM83A-AS1 was up-regulated in human LUAD tissues and closely associated with tumor size and lymph node metastasis [26]. High FAM83A-AS1 expression levels are positively correlated with poor prognosis [26,27]. Liu et al. used genome-scale analysis to demonstrate that LINC00941 expression correlates with tumor depth and distant metastasis, and might play an essential role in the regulation of metastasis and cancer cell proliferation [28]. However, there is few research to report the effect of other four lncRNAs, AC020915.2, AC245595.1, AL606834.1, and AC026369.3. For further exploring the role of the lncRNA signature, we performed pathway enrichment analysis to identify the potential biological functions. We found that apart from immune response and immune system process, the most highly enriched pathways were involved in ubiquitin mediated proteolysis and signal sequence binding.

Ubiquitin is a stable protein that acts as a targeting signal for important events in the cell, including proteasome and ribosomal function and cellular signaling [29]. Studies indicate that ubiquitin signaling plays a vital role in tumorigenesis and immune regulation [30,31]. Ubiquitin ligases can regulate the stability of oncogenes or tumor suppressors and deubiquitinate enzymes in cancer through the NF-κB signaling pathway [32] to further promote cancer development, inhibit apoptosis, and promote inflammation. Aberrant NF-κB activation is correlated with the development of lung cancer [33]. Furthermore, ubiquitin-mediated protein modification functions through signal sequence binding and ubiquitin often acts as a targeting signal for cellular events [29]. Ubiquitination is crucial in the immune T cell and B cell signaling pathways and the TNF signaling cascade, which are controlled by ubiquitin enzymes [34]. Our enrichment analysis indicates that the ubiquitin pathway has a potential role in the immune regulation of LUAD.

Immune cells are critical factors in the immune response and tumor-infiltrating lymphocytes are often implicated in the anti-tumor response to lung tumorigenesis [35]. LncRNAs are active participants in the process of immune-cell development, including hematopoietic development, myeloid differentiation, inflammatory responses, and T-cell activation [36]. Among the common immune cells, T cells are the most abundant cell type in NSCLC tumors, and account for 47% of all immune cells [37]. B cells are regarded as the second most common cells, which account for 35% [38]. The number of tumor infiltrating B cells is associated with survival [39]. Our results show that tumor-infiltrating B lymphocytes (TIBs) participate in LUAD immunity and high B cell infiltration predicts a better survival in patients with LUAD, which is consistent with the results of previous studies [40]. TIBs exist in the tumor microenvironment and exert functions in tumor development. TIBs play a vital functional role in immunity by secreting tumor-specific antibodies and promoting T-cell responses [41].

Well-controlled B lymphocyte signaling is necessary to exert an appropriate immune response and avert an excess of reactions against self-antigens following stimulation [31]. Ubiquitination-mediated B-cell regulation and signaling control are critical for tumor development, and ensures a tightly regulated humoral immune response [42]. Studies show that lncRNA exerts its function by reacting with RNA-binding proteins (RBPs) to enhance its interaction with the ubiquitin E3 ligase-TrCP1, leading to ubiquitination and degradation of RBPs [30]. The above findings indicate that lncRNAs potentially regulate the ubiquitination process in B cells.

Immunotherapy in NSCLC approaches have focused on PD-1 receptor and cytotoxic T-lymphocyte antigen-4 (CTLA-4) checkpoint receptors. The immunotherapeutic targets work by down-regulating T-cell activation and proliferation. Monoclonal antibodies directed against PD-1, such as Nivolumab and Pembrolizumab, have been approved for use in treating patients with advanced NSCLC [4]. TIBs could express Bruton tyrosine kinase (BTK) and are targetable with a BTK inhibitor. BTK inhibitors, such as Ibrutinib and other CXCL13 and CD20 antibodies [43] could reduce tumor progression in B-cell leukemias [44]. In the anti-leukemia immune responses, B cell-based immunotherapy inhibits immunosuppressive pathways and promotes the activity of cytotoxic T cells. Whereas, the role of B cell-mediated immune therapy in solid tumors is not yet established. Given the translational ability of B cell-based immunotherapeutic strategies for LUAD, B cell-related targeting medicine has gained traction [45].

In summary, we postulate that the six immune-related lncRNAs identified here could work together to affect TIBs and B cell signaling by regulating the function of ubiquitin. These immune-related lncRNAs act as a valid prognosis biomarker for LUAD. Whereas, due to the limitations of GEO data, more further researches should be conducted to validate the exact roles of six lncRNAs signature on LUAD prognosis in future. Further exploration of B-cell signaling within the tumor microenvironment and the regulation between ubiquitin and lncRNAs will help to elucidate LUAD pathogenesis and improve therapeutic strategies in the future.

## Conclusions

LUAD occurrence and development are complex processes involving interactions between tumor and immune cells. Immune-related lncRNAs in tumor-infiltrating immune cells play a key role in LUAD promotion or inhibition. The identification of an immune-related lncRNA signature provides a vital clinical tool to improve prognosis prediction and can help identify new targets for LUAD treatment.

## Supplementary Material

Supplementary Figure S1-S2

Supplementary Materials

## Data Availability

The original data in this research could be obtained from public database. The transcriptome and clinical data of LUAD were obtained from TCGA database (https://tcga-data.nci.nih.gov/tcga/). The immune-related genes were downloaded from MsigDB database (https://www.gsea-msigdb.org/gsea/msigdb), ImmPort (https://www.immport.org/home) and Immunome Database (http://hp580.angis.org.au/tagbase/gutentag/). The sequence information of lncRNAs was recorded by Ensembl database (https://asia.ensembl.org/index.html). The lncRNAs interacting proteins were estimated in catRAPID database (http://service.tartaglialab.com/page/catrapid_group). Other data that were used to support the findings of the present study are included within the supplementary material. The data used to support the findings of this study are available from the corresponding author upon request.

## Abbreviations

BTK: Bruton tyrosine kinase
CI: confidence interval
CTLA-4: cytotoxic T-lymphocyte antigen-4
HR: hazard ratio
LUAD: adenocarcinoma of lung
NSCLC: non-small cell lung cancer
OS: overall survival
PD-1: programmed death-1
RBP: RNA-binding protein
TIB: tumor-infiltrating B lymphocyte
