# Supplementary material for: Identification of an immune-related six-long noncoding RNA signature as a novel prognosis biomarker for adenocarcinoma of lung
Source: Biosci Rep. 2021 Jan 7;41(1):BSR20202444. doi: 10.1042/BSR20202444 (PMC7791552; doi:10.1042/BSR20202444)
Supplement: Supplementary Figure S1-S2 [file BSR-2020-2444_supp.pdf]

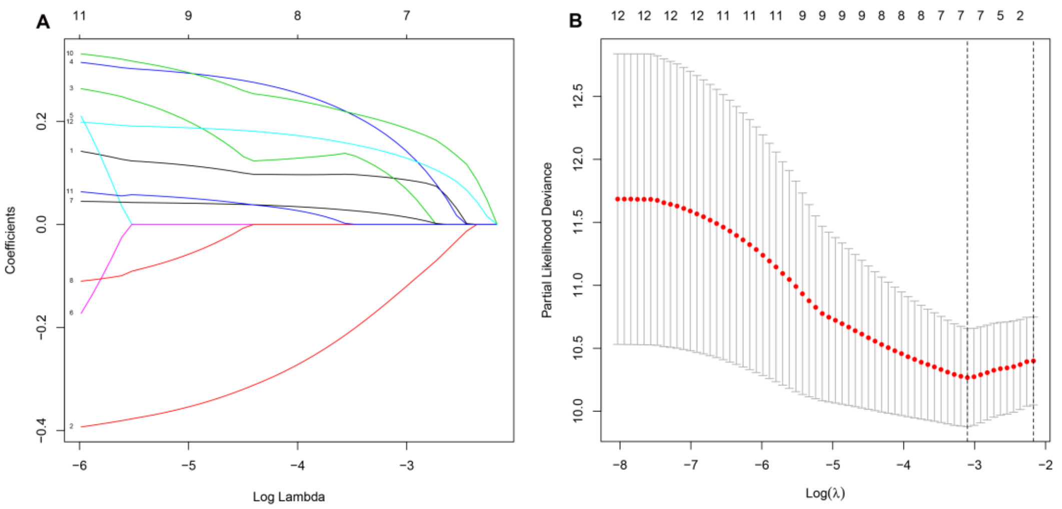

Supplementary Figure 1. LASSO and likelihood deviance plot. LASSO coefficient profiles**(A)** and a partial likelihood deviance plot**(B).**

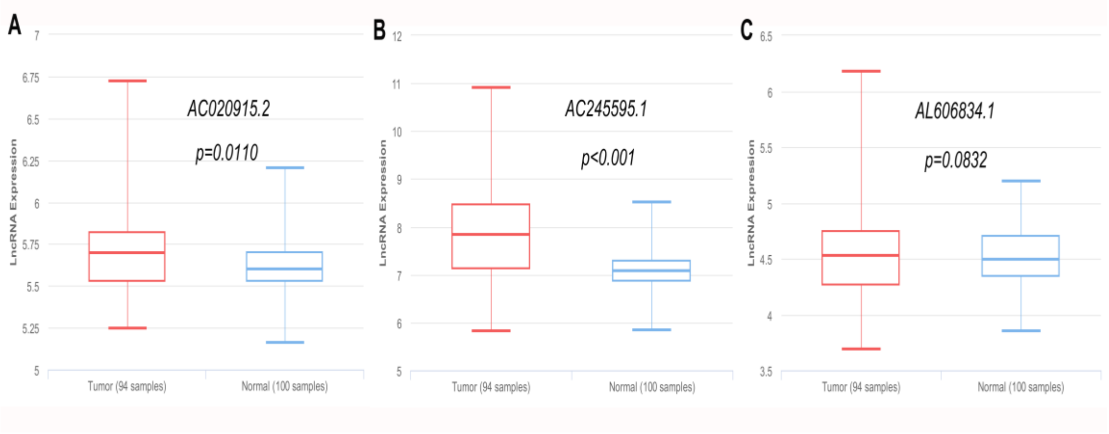

Supplementary Figure 2. The expressions of lncRNAAC020915.2 (A), AC245595.1 (B), AL606834.1 (C) in LUAD patients by LnCAR database.
