# Supplementary material for: Identification of an immune-related six-long noncoding RNA signature as a novel prognosis biomarker for adenocarcinoma of lung
Source: Biosci Rep. 2021 Jan 7;41(1):BSR20202444. doi: 10.1042/BSR20202444 (PMC7791552; doi:10.1042/BSR20202444)
Supplement: Supplementary Materials [file BSR-2020-2444_supp1.zip › BSR-2020-2444_suppST1.docx]

**Table. The sequence information of three lncRNAs (AC020915.2, AC245595.1,** **AL606834.1).**

| **AC020915.2** | >ENST00000597230.2 bp: 2708  Ensembl Transcript chromosome:GRCh38:19:1:58617616:1 |
| --- | --- |
|  | AGGCTGGTGGGGGCGGTGTTGCTCGGGGCTGCGATGCCCCTTAGGTTGCGGGCAACCAGAGGCCTCACTCGTGGGCAGGAAGCAAGGGCGGACCCCGAGAGATGACTGATTGTGGGGGTGGGGACCGTCTGCTCCTCCTAATGGCTGGGGACCGTGCACGTCTGCGCGGGCCGGACCTGCTCTTCTCGGGAACTTGCGCGGGAGGAGGCCCGGAGCTGCGCCGCTTTACCCTCTGCTCCCCGGTGCCTAAAACGCACACGCATCAAAGTCACAGGACGAGTTAGAGGAGAAAGGCAGAGGGAGATTTGAAACACAGAGGAGGTGATGAGAAGACACAGCAGAAGATATCTGAAGACTGGAAAGAATGCTGCAACCAACAGGAGAAGGAAAAGACAGAAATGTGTTTTCCATTTGGAGCATCCAGAGGGAGCACAACCATATCAACACCTTGATTTTGATCCAGTGACATTGATTTTGGACTTGTGGCCTCCAGAACTTTTAGAGAATAAATTTGCATTGTTTTAAGCCAACAGGTTGGTGGAAATTTTTTACAGCAGCCACAGGAAGCAAACACAGACTCCTAACTGGTGCTTAGTCCTAGAATATCAGTTTCTGTTCCACTTCTGGATCTCATAATTGCATCTCAGACATCAACACTGACTTCATATTCTTAATGCACCAATTCTACCATTTCTTCAGGCTGCAAACAGAGAAAAACTTATTTAAGCATTCCAGAATTGACCCAATAGAAGACTCTCCAGAAAAGACTGCATGGTCTCTGAAGCAGCATGATCAGCATGACTGGCTGCATCTGGGCTCCACCGTTTGCCACTTCAGACAGGTCCTTAGCCTCTTAGAGGTTCATTTTCCTTGCTGTAGCCCGGAGTAAGAATGTTTAGCCTACAAGATTTATTTTGAGGCATCAATGAATCACTATGTGAGCCTCACACAGTGTCTGGCACTGATATTACAAAATATGGTTGATCTTGCCATCCTTGTTATTGTCCTGGTGGGGATTCCTCTCTGTTGAAGATACGGAGCCTCTAAAAATGAGTGGCAGAGGCTCCCAGCTCAGGATCCTTGTCCAGGTCCTAAAGATCCTCCACTCTGCTTGTCACTCTGTTCATTATGTTTGCATGGCCACCACGGTCAACACTACTGCGGCCTACCTCAAGTTCTCATGCCTCACTCTCTGAGCTCATGAGGACACAAGGATCCTTTGGCAACACATTAACTACACTCTGTGGCCATCCCTTGACTTCTGAGAGGATGTTTCCCTCCTCTCAATCAAAAGCTCTCTTCCCTCCTGGGCAAGATCCTTGCATGCACCCAGCACCCTCACACCTGGTGCCCTCACACCCAGCACTCTCACACGTGGTGCCCTCACACCCAGCACCCTCACACCCAGCACCCTCACACCTGGTGCCCTCACACCCAGCACCCTCACACCTGGTGCCCTCACACCCAGCACCCTCACACCTGGTGCCCTCGCACCCAGCACCCTCACACCTGGTGCCCTCGCACCCAGCACCCTCACACCTGGTGCCCTCGCAACCAGCACCCTCACACCTGGTGCCCTCACACCCAGTGCCCTCACACCCAGCACTCTCACACTTGCACTGTCCTCATCCCAACTCTGTGGTTCCTCTGGGGAATCACTGTGGAATCCCTCATGGGAGGATGTTTTCAAAAGAAACAACAGCCCAATGAAGGGTATTTGAGGATGAGAAGCAGGAAAGAAGTCAGTGGGTGGCTGAGTTGTGTCCAGGTTTTGCGTGGCCATGGAAAATCAGGTGAAGGACATTAAATATCTTGCAGTGGCTTCTCCATTAGGAGCATGAAATGAGGAAAGGGGCTGTCCTCACAGGCCAGCCAAGAGGGATGCCTATGCCAAACAGAAAGACTGACAGTAAACAGAATCCAGGATGACATGACTGAGGTATGGTGTGCAGGGCATGGCCCAGGGAGGAGGGTAGATAAGTAAGCCTGGGTGCACCTTCAGAGGAAGAAATAACAGAGGCTGCAGGCGAGAGAGTTACAAGGAAGGAGGAGCAGGAAGACACGAGAAAAATTCAGATAAACTCAACATCTCCAGCCTGGGGGAAACGTGAGAGGGTGGTATGAAGATACACACGATGCATGTTGCTTAGTGAAAGCAGCCAGTCGGAAAAGGCTACGTAGTGAATGACCCCAACTATGACATTCTGAAAAAGGCAAAACTATGGAGACAGGATAAAGATCAGTGGTTGCCAGTGAGGCAGGAGAATAGGGTCTGGGGGCAGGGAGCCTAAGGCTGATTCATGCTGACTTCATGAACGGAATCAAAAGGAAAACCCTAAATTTCCATGCCCACGTAACAAAAGGATCAGAGGCTACTCCCTTTGCAACTCCCCCCAACTTTCTGTGTTGCAGATGAAAAATGGAAAGTGCCTCGGATTGGTCCCCTCCTGCAACCAGTCAGACATCTGCATAGAGCGTAACTTTGTAACTTCACTTCAGCCTCTGCTTGGTCACCTTCCACAACCAATCAGACTGGTCATGGGCCACTATTTCATAGGGTGTAAACCAAGTGACCAATGGGAAACATCTAGAGGGTATTTAAACCCCAGAAAATTCTGCGACCAGTGCTTTTGAGCCTTGCTTGGGCCTGCTCCCACTCTGTGAAGTGCATTTTTGTTTCAATACAGCTATGCTTTTGTTGCTTCTTT |
| **AC245595.1** | >ENST00000611488.1 bp: 1144  Ensembl Transcript chromosome:GRCh38:1:1:248956422:1 |
|  | AGGAGGCTTGGAGTACCCATAATACAGTGAGCCCACCTTCCTGATCCCCAGACATTTCAGGAGGTCGGGAAATTTTTAAACCCAGGCAGCTTCCTGGCAGTGCCATTTGGAGCATCAAAGTGGGCCATGGGTCTGGATTTCCAGGAAAGCGGAGACCTCGAGGTGCAGGACTGTCGGGGCGAGGTGGCCGAGGCAGGTCAAAGCTGAAAAGTGGAATCGGAGCTGTTGTATTGCCTGGGGTGTCTACTGCAGATATTTCATCAAATAAGGATGATGAAGAAAACTCTGTGCTCGATATGGTTGTGTTGTTTTCTAGCAGTGACAAATTCACTTTGAATCAGGATACATGTGTAGTTTGTGGCAGTTTTGGCCAAGGAGCAGAAGGAAGATTACTTGCCTGTTCTCAGTGTGGTCAGTGTTACCATCCATACTGTGTCAGTATTAAGATCACTAAAGTGGTTCTTAGCAAAGGTTGGAGGTGTCTTGAGTGCACTGTGTGTGAGGCCTGTGGGAAGGCAACTGACCCAGGAAGATTCCTGCTGTGTGATGATTGTGACATAAGTTATCACACCTACTGCCTAGACCCTCCATTGCAGACAGTTCCCAAAGGAGACTGGAAGTGCAAATGGTGTGTTTGGTGCAGACACTGTGGAGCAACATCTGCAGGTCTAAGATGTGAATGGCAGAACAATTACACACAGTGCGCTCCTTGTGCAAGTTTATCTTCCTGTCCAGTCTGCTATTGAAACTATAGAGAAGAAGATCTTATTCTGCAATGAAGACAATGTGATAGATGGATGCATGCAGTTCGTCAGAACTTAAATACTGAGGAAGAAGTGGAAAATGTAGCAGACATTGGTTTTGATTGTAACATGTGCAGACCCTATATGCCTGCGTCTAATGTGCCTTCCTCAGACTGCTGTGGATCTTCACTTGTAGCACAAATTGTCACAAAAGTAAAAGAGCTAACCCACCCAAGACTTATACCCAGGATGGTGTGTGTTTGACTGAATCAGGGAAGACTCAGTTACAGAGCCTCACAGTTACAGTTCCAAGAAGAAAACTGTCAAAACCAAAACTGAAATTGAAGATTATAAATCAGAATAGCGTGGCCGTCCTTCAGACCCCTCCAGACATCCAATCAGAACATTCAAGGGATGGTGATATGGATGATAGTCGAGGTAATACTAATTTATT |
| **AL606834.1** | >ENST00000602954.1 bp: 668  Ensembl Transcript chromosome:GRCh38:14:1:107043718:1 |
|  | ACAGTTTTCTGTTCTTTTTACTTGAATGATTTTCTTCATGTTGTATACTTTTTATGAGTGGTTTTAATGACTGCGTAATCTTCCATTTGGTGCATATTCAGTGGTTAATGAGGACTTGACCTATACTTCAGAAATTTGGAAAGAGGAGAAGTGGGACACGCCCACATTAATGGGACTTGGCCACTAACGGGGTTTGAAGGACAAAGGAGAGGGAAGAGTCAGATAGGATTAGCACAGCACATGACTGCCTAAGAGATGCTCACAAAAATAAATGGCTGAATGAGTGAATGAACCCAGCGGTTTTAAATCTGGAGGCTTAAGACAGTTTTTGGTAGCTGAGGGAGAACAGAAATAGCTATGTTTACTGTTAATATAGAATATGTGTTATTCTTTGCGTTCACCTTTCCCACCTATATTGTAGACGTCACTGATAGCTTGTAATACTTTTCTACCTGAGGTTTAAGTATGTAAATATGAGGAATTAATAACAAGTTAAACTCTTGTTTTGAAAATCTAATTTGCCAAATATAATTGAGGAAATACTATAAGAACTAAATCTCTACTGATCCTCTCTTAGGAGCAATTTGAGATAATTTTTCTAAGTTTAAAGACAGACTTTAAAGCAACTTTCCAGTCACAACTTCAAAATAAAGTACAAGTTAATTGCA |
